# Work community factors, occupational well‐being and work ability in home care: A structural equation modelling

**DOI:** 10.1002/nop2.1032

**Published:** 2021-08-15

**Authors:** Anneli Vauhkonen, Terhi Saaranen, Kirsi Honkalampi, Susanna Järvelin‐Pasanen, Saana Kupari, Mika P. Tarvainen, Merja Perkiö‐Mäkelä, Kimmo Räsänen, Tuula Oksanen

**Affiliations:** ^1^ Department of Nursing Science Faculty of Health Sciences University of Eastern Finland Kuopio Finland; ^2^ School of Educational Sciences and Psychology Philosophical Faculty University of Eastern Finland Joensuu Finland; ^3^ School of Medicine Faculty of Health Sciences University of Eastern Finland Kuopio Finland; ^4^ Department of Applied Physics Faculty of Science and Forestry University of Eastern Finland Kuopio Finland; ^5^ Department of Clinical Physiology and Nuclear Medicine Kuopio University Hospital Finland; ^6^ Institute of Public Health and Clinical Nutrition School of Medicine Faculty of Health Sciences University of Eastern Finland Kuopio Finland

**Keywords:** factors, home care, occupational well‐being, structural equation modelling, work ability, work community

## Abstract

**Aim:**

To examine how work community factors are related to occupational well‐being and work ability, and how occupational well‐being is related to work ability.

**Design:**

A cross‐sectional study was conducted among home care workers in one municipality in Finland.

**Methods:**

A self‐administered survey on work and well‐being was filled out by 167 employees working two shifts in 2019. Structural equation modelling was used to analyse the association between work community factors, occupational well‐being and work ability.

**Results:**

The only work community factor directly affecting *Occupational well‐being* was *Information and work organization*; the effect of the other two factors, *Social support* and *Influence on work shifts*, was indirect. All work community factors indirectly affected *Work ability*. Home care should emphasize information provision and work organization with optimal time use. This requires social support, a well‐functioning work atmosphere and providing employees with opportunities for influence and participation.

## 1 INTRODUCTION

The social benefits of occupational well‐being and work ability have been rightly demonstrated, as they play a key role in organizational performance, sick leave rates, customer satisfaction, lower employee turnover and intention to retire (Ilmarinen et al., 2008; Prakash et al., 2019; Finnish Institute of Occupational Health, 2020). The European Union has emphasized that rapid changes occurring in working life change the needs and means related to the development of occupational well‐being and work ability in different professions (EU‐OSHA, 2018). These changes are tangible in home care for older people, because the number of people aged 65 and over is increasing in every EU member state (Eurostat, 2020). At the same time, the proportion of workers aged 50 or over has increased, and the proportion of younger workers has decreased (Eurofound, 2017). The employees in the healthcare sector were already experiencing higher levels of work intensity before the coronavirus pandemic (Eurofound, 2017). The ongoing coronavirus pandemic is posing an even greater challenge to healthcare employees worldwide. The employees have an increased risk for developing a COVID‐19 infection, and the virus has led to an increase in their work‐related psychological stress and anxiety (Nguyen et al., 2020; Temsah et al., 2020). Especially in home care whose clients are in a high‐risk category due to their age, the psychosocial workload of employees may increase.

Many countries have transitioned to using a strategy that involves prioritizing home‐based care (Genet et al., 2011; Spasova et al., 2018). In Finland, the structure of services for older people has shifted from institutional care to home‐based services with the aim of ensuring a better quality of life, equality, increased coordination and cost‐effectiveness of services for older people (Kalliomaa‐Puha & Kangas, 2018; Noro & Karppanen, 2019; The Ministry of Social Affairs and Health, 2018). The number of people who need a lot of support and services in home care has increased throughout the country (Kehusmaa et al., 2018; Noro & Karppanen, 2019). In Finland, almost half (44%) of home care clients use services that involve considerably diverse support and help the clients cope with their everyday lives (Finnish Institute for Health & Welfare, 2020; Kehusmaa et al., 2018).

In Finland, most home care workers are practical nurses, and they form the largest professional group in the social and healthcare sector in Finland. They have vocational qualifications in social and health care (180 ECVET competence points) whereas other caregivers in home care have a shorter education. In this study, all occupational groups working in home care are referred to as home care workers. Their work in home care includes providing clients with basic and medical care, and supporting the client's ability to function, health, well‐being, and independence in daily activities.

Simultaneously as the provision of home care has increased, the occupational well‐being of home care workers has decreased as their workload has grown (Ruotsalainen et al., 2020). Home care workers also experience work‐related stress (Muramatsu et al., 2019; Otto et al., 2019; Ruotsalainen et al., 2020), high levels of time pressure (Otto et al., 2019; Ruotsalainen et al., 2020) and diverse stressors related to specific clients and challenges of work community such as a lack of training and information, adequate work organization and support from supervisors (Muramatsu et al., 2019; Ruotsalainen et al., 2020). These challenges related to the work community make employees feel exhausted which has led to an increase in sick leaves, further adding to the workload for those still at work and employees' worries about coping at work (Ruotsalainen et al., 2020; Vehko et al., 2018). In light of these situations, it is important to promote home care workers' occupational well‐being and work ability, avoid staff overload and maintain the availability of home care workers. Maintaining work ability can be considered an important social and economic objective to address the challenges related to an ageing population and reduce early withdrawal from work (Eurofound, 2017; Ilmarinen, 2019).

## 2 BACKGROUND

In the scientific literature, the concept of occupational well‐being has not yet been properly established (Buffet et al., 2013; Schulte & Vainio, 2010). Instead, occupational well‐being has been described with the terms job satisfaction, intent to stay working and work engagement, resources and work‐related stress (e.g. Hirschl & Gondim, 2020; Kvist et al., 2012; Ruotsalainen et al., 2020). These concepts have often been viewed from the perspective of individual workers and their work, the work community, and the work organization (Arian et al., 2018; Cotton & Hart, 2003; Hirschl & Gondim, 2020; Schulte & Vainio, 2010). Recently, the focus has shifted on empowerment, and occupational well‐being has been defined as an empowerment process for the individual and the community (Bartels et al., 2019; Laine, Saaranen, et al., 2018; Laine, Tossavainen, et al., 2018; Mäkiniemi et al., 2014). As a result, the aim has been to balance the resources and workload factors of the individual and the work community in relation to work requirements. In this context, resource and workload factors can be viewed from the perspective of the employee, working conditions, work community and professional competence (Laine, Saaranen, et al., 2018; Laine, Tossavainen, et al., 2018).

Occupational well‐being and work ability are closely related concepts, containing the same individual and organizational elements (Cotton & Hart, 2003; Ilmarinen et al., 2008; Tengland, 2010; Laine, Saaranen, et al., 2018; Laine, Tossavainen, et al., 2018). Similarly as with occupational well‐being, there are several definitions of work ability (see e.g. Ilmarinen, 2009; Tengland, 2010). Work ability is often only perceived as an individual resource related to health, competence, values, attitudes and motivation in relation to job requirements, such as managing work (Ilmarinen et al., 2008; Tengland, 2010). The present study perceives work ability as a wider concept that comprises health and functional capacity, professional experience, values, attitudes, work environment and work demands, and that is also related to the work organization, work community and society (Ilmarinen et al., 2008). This theoretical framework for work ability called *the house of work ability* model consists of the resources of the individual worker and work‐related factors, and the social environment. Therefore, good work ability is fostered by a balance between the individual worker's resources and work factors. (Ilmarinen et al., 2008, 2015, Ilmarinen, 2019).

Several studies have found evidence that work community factors, such as social support, employees' opportunities to influence their work, information and organization in the work community, are related to work ability and occupational well‐being (e.g. Bartels et al., 2019; Van der Heijden et al., 2017; Hirschl & Gondim, 2020; Laine, Saaranen, et al., 2018; Laine, Tossavainen, et al., 2018). Social support has been perceived as one of the dimensions of social capital (Nieminen et al., 2013). Putnam (1995) defines social capital as networks, norms, and trust, which enable participants to act together more effectively to pursue shared objectives. According to Hyyppä (2010), communality and social capital can be considered as parallel concepts. Communality and social capital emerge from the trust, open communication, interaction, participation and learning of the members of the organization (Pärnänen, 2006; Saaranen et al., 2015). High social capital is associated with good organization of work at the workplace (Pärnänen, 2006), and social capital can be seen as a determinant of health (Hyyppä, 2010; Kouvonen et al., 2008; Oksanen et al., 2008). These work community factors and resources are also fundamental in work‐related theoretical models, such as the Demand‐Control‐Support model and Job Demands‐Resources model (see Demerouti et al., 2001; Johnson & Hall, 1988).

Social support has been identified as a resource at work, which may reduce work‐related distress (Van der Heijden et al., 2017; Hirschl & Gondim, 2020) and serve as a buffer against the association of work‐related stressors and occupational well‐being (Hirschl & Gondim, 2020). According to Van der Heijden et al. (2017), the quality of leadership and social support from leaders and colleagues have been found to be positively associated with the occupational well‐being of the nurses (incl. home care) and reduce psychological distress. Recognition by supervisors and organization has also been found to promote job satisfaction among home care workers (Jang et al., 2017). By contrast, a lack of social support and low decision‐making power have been reported to negatively influence employee well‐being (Hirschl & Gondim, 2020). Moreover, low social support has been found to relate to an increased probability of poor work ability (Leijon et al., 2017).

Employees' opportunities to influence their work have been found to have a positive relationship with occupational well‐being and work ability (Galatsch et al., 2013; Hirschl & Gondim, 2020; Leijon et al., 2017; Nelson et al., 2014; Van Poel et al., 2020). According to Nelson et al. (2014), authentic leadership was related to the psychological well‐being of nurses. Decision authority was related to an increased probability of poor work ability (Leijon et al., 2017). Worker autonomy can reduce the impact of stressors on occupational well‐being (Hirschl & Gondim, 2020). Galatsch et al. (2013) found a substantial decrease of work ability and health among nurses whose requests for shift schedule changes were not fulfilled. Information, work management and time use have been found to be associated with the well‐being of the work community (Laine, Saaranen, et al., 2018). Time pressure or heavy mental workload regarded as high job demands was associated with nurses' poorer perceptions of their work ability (Van Poel et al., 2020). According to Ruotsalainen et al. (2020), work interruptions and time pressures were associated with higher perceived stress in the work of Finnish home care workers.

Several previous studies have concerned the relationship between work community factors and occupational well‐being (see e.g. Van der Heijden et al., 2017; Hirschl & Gondim, 2020; Laine, Saaranen, et al., 2018; Laine, Tossavainen, et al., 2018; Nelson et al., 2014) and work ability (Galatsch et al., 2013; Leijon et al., 2017; Van Poel et al., 2020). Research from the perspective of home care workers is very limited (see e.g. Jang et al., 2017; Ruotsalainen et al., 2020). Previous studies have involved creating occupational well‐being or work ability models to examine, among other things, the determinants of teachers' work ability (Alcantara et al., 2019), four aspects of occupational well‐being (working conditions, work community, worker and work, and professional competence) in the school context (Laine, Tossavainen, et al., 2018; Saaranen et al., 2007), eudaimonic and hedonic workplace well‐being (Bartels et al., 2019), and work ability promotion and measurement evaluation have been examined with *the house of work ability* model (see e.g. Ilmarinen, 2019; Ilmarinen et al., 2015). However, to our knowledge, the relationship between these concepts (occupational well‐being and work ability) has not been studied together with the perspective of work community factors in the context of home care. Boschman and others (2018) have studied intrapersonal fluctuations in well‐being connected to task‐specific work ability through multilevel modelling. Although their study was focused on the non‐work‐related aspects of well‐being, based on their findings, poorer well‐being in the context of work and social contacts was related to poorer task‐specific work ability. (Boschman et al., 2018.) Work ability can be seen as a key asset in employees' working life (Ilmarinen, 2009, 2019). As employees need work ability to cope at work and occupational well‐being promotes work ability, (Ilmarinen, 2009, 2019; Schulte & Vainio, 2010), work ability is considered as an outcome concept in this study.

The aim of this study was to examine how work community factors are related to occupational well‐being and work ability, and how occupational well‐being is related to work ability.

The research question of this study is:

1. What is the relationship of work community factors (*Social support, Influence on work shifts, Information and work organization*) to *Occupational well‐being* and *Work ability* and, furthermore, what is the relationship of *Occupational well‐being* to *Work ability*?

**FIGURE 1:**
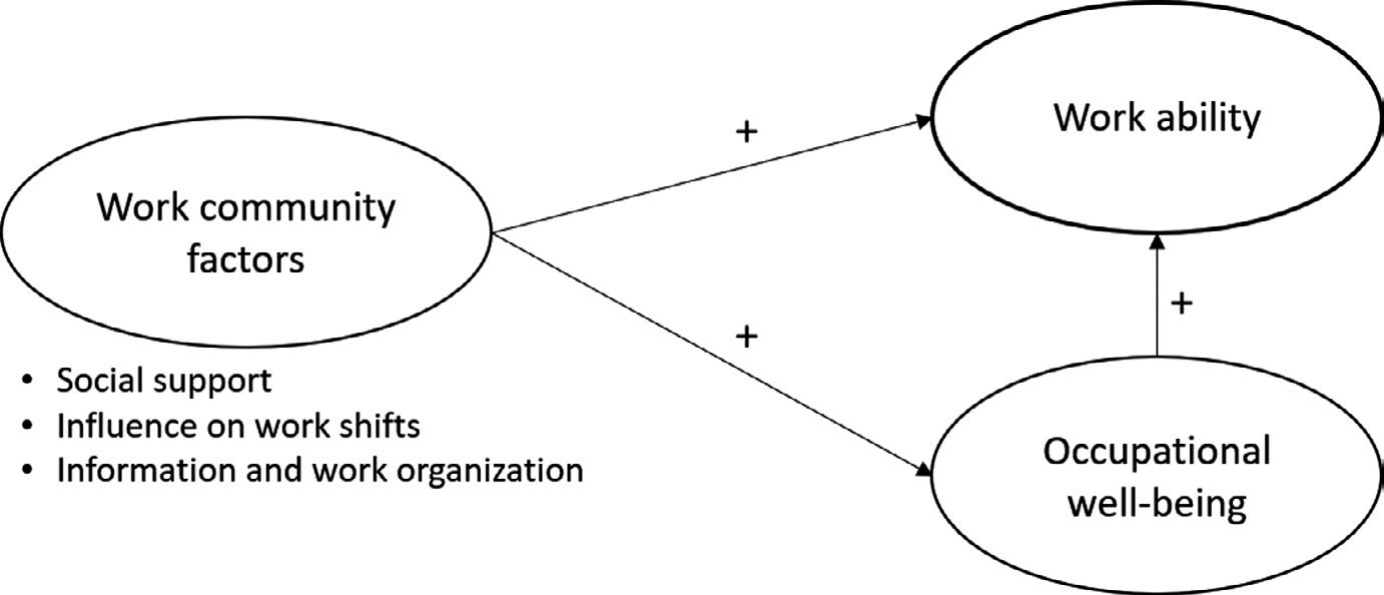
Hypothetical model of the connections between work community factors, work ability, and occupational well‐being

The hypotheses were based on previous research (e.g. Boschman et al., 2018; Van der Heijden et al., 2017; Hirschl & Gondim, 2020; Ilmarinen, 2019; Laine, Tossavainen, et al., 2018; Van Poel et al., 2020) as follows (Figure 1):

1. The work community factors affect *Work ability* or/and *Occupational well‐being* (direct, indirect or/and total effects)
2. *Occupational well‐being* significantly and directly affects *Work ability*

## 3 METHODS

### 3.1 Design

This cross‐sectional study was related to a research project conducted among home care workers in one municipality in Finland. Therefore, all employees comprised the eligible population of the study; every home care worker was offered an opportunity to participate in this study. The focus was on work community factors (*Social support, Influence on work shifts, Information and work organization*), *Occupational well‐being* and *Work ability*. Structural equation modelling (SEM) was used to analyse the association between work community factors, *Occupational well‐being* and *Work ability*.

### 3.2 Questionnaire and data collection

The questionnaire used in the study was developed in a multidisciplinary (psychology, occupational health, nursing science) research group and tested in an academic thesis (Hirvonen, 2018). Prior to this study, in early 2019, the questionnaire was re‐examined in a multidisciplinary study group (the above disciplines and applied physics) and tested by 11 home care workers. Based on an analysis of received feedback and pre‐testing material, the questionnaire was deemed functional. The questionnaire was sent to all home care workers working in two shifts at the home care services of one municipality in Eastern Finland (*N* = 370) in autumn 2019. A total of 167 of them responded to the survey (response rate 45%) in 2019.

*Occupational well‐being* was measured using two items: personal occupational well‐being in this profession compared with the best possible level and general occupational well‐being in the respondents' work community (Likert scale 1–5, 1 = very poor, 5 = very good) (Laine, Tossavainen, et al., 2018; Saaranen et al., 2007, 2015). *Work ability* was considered to consist of three measures; work ability score (scale 0–10, 0 = full work disability, 10 = work ability at its best), work ability in relation to the physical demands of the work and work ability in relation to the mental demands of the work (Likert scale 1–5, 1 = very poor, 5 = very good). (Tuomi et al., 1998; Gould et al., 2008). The work community aspect of occupational well‐being was measured using 11 Likert scale items (Likert scale 1–5, 1 = very poor, 5 = very good) (adapted from Laine, Tossavainen, et al., 2018; Saaranen et al., 2007, 2015).

### 3.3 Data analysis

The data were analysed using IBM SPSS statistics 27, AMOS 27 and Stat Tool Package by James Gaskin (Excel). First descriptive analysis (frequency, percentage, mean, standard deviation (SD), minimum, maximum) was used to describe the demographics, occupational well‐being, work ability and work community. Subsequently, an explorative factor analysis (EFA) was performed with maximum likelihood extraction and varimax rotation method to form unifying dimensions from the 11 items measuring the work community aspect of occupational well‐being. The reliability of the factors was examined using the Cronbach's alpha coefficient. Based on preliminary EFA, two items were excluded because of relatively low communalities and factor loadings, and they did not fit into the theoretical structure. Therefore, the analysis was repeated with 9 items (Table 1). Kaiser–Meyer–Olkin (KMO) measure 0.845 indicated good sampling adequacy for the analysis. Based on the scree plot, items loaded on three factors, which formed the work community factors and were titled: *Social support, Influence in work shifts*, and *Information and work organization*. Mean variables were formulated based on EFA to describe the state of the work community. (Field, 2018.)

**TABLE 1:** Work community factors, items, factor loadings, % of variance and Cronbach's alphas of work community factors

Work community factors	Factor loadings	% of variance	Cronbach's alpha
Social support			
Personal relationships between workers at my workplace are fine	0.822	24.017	0.875
I get help and support from my colleagues when needed	0.794		
There is a spirit of “fair play” at my workplace, and there is no harassment of workers	0.792		
Influence on work shifts			
There are no problems with shift arrangements in my work	0.595	24.618	0.850
I can contribute to solving problems related to shift arrangements	0.619		
I can influence which shift I am working	0.774		
My personal wishes are taken into account well in preparing the shift list	0.782		
Information and work organization			
Organization of work and time use are good in my work community	0.771	17.956	0.836
Sufficient information has been provided about changes in the work community	0.767		

Structural equation modelling was performed to examine the connections between home care workers' work community factors and perceived *Occupational well‐being* and *Work ability*. Six values were missing at random from the data and were replaced with the mean of that item to perform the SEM analysis. SEM is an advanced statistical theory testing method combining factor and regression analysis and allowing the simultaneous examination of many equations. (Blunch, 2013.) The SEM was conducted using a two‐step approach; 1. Creating a measurement model with the variables and items mentioned above and testing the factor structure in a confirmatory factor analysis (CFA), 2. Constructing the structural equation model from the measured factors. The normality of the data was tested by examining kurtosis statistics, because SEM is based on the covariance structure. A critical ratio of the multivariate kurtosis value (10.532) indicated non‐normality. Therefore, bootstrapping was used together with the maximum likelihood estimation method to improve the chi‐squared test of model fit (Bollen‐Stine bootstrap method) and to examine the effects between the variables (Walker & Smith, 2017).

The hypothesized factor structure was tested by CFA. The following criteria were used to evaluate the measurement model and SEM: *χ* ^2^ with the degrees of freedom (DF) and Bollen–Stine bootstrap *p*‐value (>.05), root mean square error of approximation (RMSEA) (<0.08), standardized root mean square residual (SRMR) (<0.08) and comparative fit index (CFI) (≥0.95). (Blunch, 2013; Byrne, 2010; Schreiber et al., 2006; Walker & Smith, 2017.) In addition, the validity of the measurement model was tested using composite reliability (CR) (>0.70), average variance extracted (AVE) (>0.50), and Fornell–Larcker (FL) test. Based on the modification indices, two error terms of *Influence on work shifts* factor were connected. The preliminary model had validity concerns with the *work ability* latent variable. Therefore, two single items, work ability in relation to physical and mental demands of the work, were combined into a sum variable (Work ability in relation to the demands of the job, scale 2–10) reducing the correlation between the factors of *Occupational well‐being* and *Work ability*. Hence, CFA confirmed the modified hypothesized factor structure with the following model fit indices: *χ* ^2^ = 70.988, DF = 54, *p*‐value = .305, RMSEA = 0.044, SRMR = 0.0514, CFI = 0.985 and validity measures: AVE = 0.554–0.718, CR = 0.774–0.836 and square root of AVE was greater than inter‐construct correlations. The hypothesized model (Figure 1) was evaluated by SEM. The relationships between the factor variables were tested, and direct, indirect and total effects were examined. The model was adjusted by deleting non‐significant effects. (Blunch, 2013; Byrne, 2010; Gaskin, 2021).

### 3.4 Ethical consideration

Research ethics committee approval was obtained from the Research Ethics Committee of the Northern Savo Hospital District (13.02.00412/2019) in spring 2019. A signed informed consent to participate in the study was collected from each participant. The participants were informed about the voluntary nature of the study and their right to withdraw from the study without consequences. The general data protection regulation (GDPR 2016/679) was followed in collecting, processing and storing the data.

## 4 RESULTS

### 4.1 Participants

Most of the study participants were women (88.6%), practical nurses (95.8%) and were married or co‐habiting (69.4%). The average age of the participants was 42, and they had been in their current position for an average of 8 years. 70% of the employees were in a permanent employment relationship. (Table 2.)

**TABLE 2:** Demographics of the study participants (*n* = 167)

Variable	*N*	%	Mean (*SD*)	Min.	Max.
Sex (*N* = 167)					
Female	148	88.6			
Male	19	11.4			
Age (*N* = 161)			41.6 (13.1)	19	64
Age group in years					
<45	88	54.7			
45≤	73	45.3			
Job title (*N* = 167)					
Practical nurse	160	95.8			
Caregiver	7	4.2			
Marital status (*N* = 167)					
Married/Co‐habiting	116	69.4			
Divorced/single/widowed	40	24.0			
Other	11	6.6			
Work experience in years					
In the current workplace (*N* = 154)			8.0 (9.0)	0.0	39.0
In shift work (*N* = 154)			12.1 (10.0)	0.4	39.0
Employment relationship (*N* = 167)					
Permanent	117	70.1			
Temporary	50	29.9			

### 4.2 Occupational well‐being, work ability and work community factors in home care

More than half of the participants (61%) rated their personal occupational well‐being as quite or very good. Correspondingly, less than half (43.7%) perceived the general occupational well‐being of their work community as quite good or very good. The participants gave their work ability an average score of 8.1 (*SD* = 1.3). Nearly three‐quarters of the participants rated their work ability in relation to the physical demands of the work (74.3%) and work ability in relation to the mental demands of the work (73.7%) as quite good or very good.

In the work community, the mean of *social support* was 4.1 (*SD* = 0.8), *influence on work shifts* 3.6 (*SD* = 0.9), and *Information and work organization* 3.0 (*SD* = 1.1). The majority of the responders felt that they received help and support from their colleagues whenever needed. Correspondingly, less than half of the participants felt that there were shortcomings in the work organization and time use of the work community. (Table 3.)

**TABLE 3:** Mean variables and the individual items of the work community, mean (scale 1–5), standard deviation, %, *N* = 164–167

	Mean (*SD*)	Totally disagree	Somewhat disagree	Neither agree nor disagree	Somewhat agree	Totally agree
Social support						
Personal relationships between workers at my workplace are fine	4.1 (0.8)	1.8	6.0	10.2	52.4	29.5
I get help and support from my colleagues when needed		1.8	4.8	1.8	40.1	51.5
There is a spirit of “fair play” at my workplace, and there is no harassment of workers		3.6	4.8	15.0	40.1	36.5
Influence on work shifts						
There are no problems with shift arrangements in my work	3.6 (0.9)	10.2	25.3	19.9	30.1	14.5
I can contribute to solving problems related to shift arrangements		2.4	13.9	19.3	44.6	19.9
I can influence which shift I am working		3.6	11.4	13.9	54.8	16.3
My personal wishes are taken into account well in preparing the shift list		1.8	10.2	13.8	46.1	28.1
Information and work organization						
Organization of work and time use are good in my work community	3.0 (1.1)	8.4	33.5	15.6	31.7	10.8
Sufficient information has been provided about changes in the work community		7.8	32.9	18.0	29.3	12.0

### 4.3 Relationships between work community factors, occupational well‐being and work ability

**FIGURE 2:**
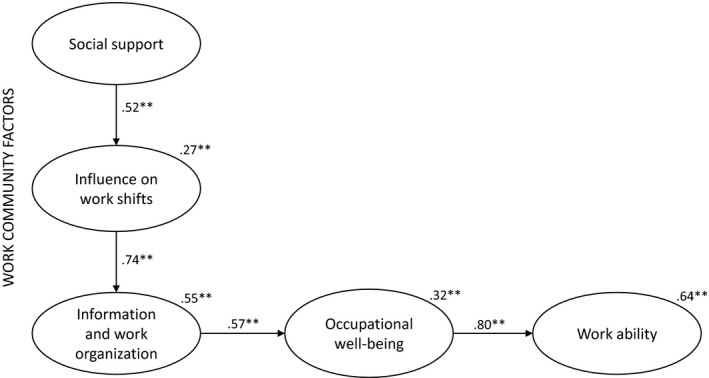
The Model of Occupational Well‐being and Work Ability of Home Care Workers: a perspective of work community factors. Standardized regression weights (value range – 1 to +1), squared multiple correlations (above the factors on the top right) describing variation explained by the model (value range 0–1), ** means *p* < .01

The model fit indices (*χ* ^2^ = 81.413, DF = 60, *p*‐value = .269, RMSEA = 0.046, SRMR = 0.0684, CFI = 0.980) indicated that the SEM model was supported by the data. *Social support* had a statistically significant direct effect on *Influence on work shifts*, but a non‐significant effect on *Information and work organization* (not shown in the Figure 2). *Influence on work shifts* had a significant direct effect on *Information and work organization*. In this model, *Information and work organization* was the only significant work community factor directly affecting *Occupational well‐being*. *Occupational well‐being* had the significant direct effect on *Work ability*. (Figure 2, Table 4).

**TABLE 4:** Estimates for structural equation model, relationships, estimates and *p*‐values

Relationship			Estimate	*p*
Standardized regression weights (standardized direct effects)				
Influence on work shifts	<‐‐‐	Social support	0.519	.001**
Information and work organization	<‐‐‐	Influence on work shifts	0.743	.001**
Occupational well‐being	<‐‐‐	Information and work organization	0.569	.001**
Work ability	<‐‐‐	Occupational well‐being	0.802	.001**
Standardized indirect effects				
Information and work organization	<‐‐‐	Social support	0.386	.001**
Occupational well‐being	<‐‐‐	Social support	0.219	.001**
Work ability	<‐‐‐	Social support	0.176	.001**
Occupational well‐being	<‐‐‐	Influence on work shifts	0.422	.001**
Work ability	<‐‐‐	Influence on work shifts	0.399	.001**
Work ability	<‐‐‐	Information and work organization	0.456	.001**
Standardized Total effects				
Influence on work shifts	<‐‐‐	Social support	0.519	.001**
Information and work organization	<‐‐‐	Social support	0.386	.001**
Occupational well‐being	<‐‐‐	Social support	0.219	.001**
Work ability	<‐‐‐	Social support	0.176	.001**
Information and work organization	<‐‐‐	Influence on work shifts	0.743	.001**
Occupational well‐being	<‐‐‐	Influence on work shifts	0.422	.001**
Work ability	<‐‐‐	Influence on work shifts	0.339	.001**
Occupational well‐being	<‐‐‐	Information and work organization	0.569	.001**
Work ability	<‐‐‐	Information and work organization	0.456	.001**
Work ability	<‐‐‐	Occupational well‐being	0.802	.001**
Squared multiple correlations				
Influence on work shifts			0.270	.001**
Information and work organization			0.551	.001**
Occupational well‐being			0.324	.001**
Work ability			0.643	.001**

Note

** means *p* < .01.

Examining the indirect and total effects, *Social support* had a significant total and indirect positive effect on all latent variables in the model so that other work community factors and *Occupational well‐being* served as positive mediators strengthening the effects. *Influence on work shifts* had a significant indirect effect on *Occupational well‐being* and *Work ability*. *Information and work organization* strengthened the positive relationship of *Influence on work shifts* to *Occupational well‐being*, and *Occupational well‐being* and *Information and work organizations* strengthened the positive effect of *Influence on work shifts* on *Work ability. Information and work organizations* had a significant indirect effect on *Work ability*. The created model explained 64% of the observed variation in the dependent variable *Work ability*. (Table 4).

## 5 DISCUSSION

This cross‐sectional study provided new evidence on how work community factors are related to occupational well‐being and work ability in the context of home care based on structural equation modelling. The model supports an idea that *Work community* factors are related to *Occupational well‐being* and *work ability* (hypothesis 1) and that *Occupational well‐being* significantly and directly affects *Work ability* (hypothesis 2). The main findings of this study were that the only significant work community factor directly affecting *Occupational well‐being* was *Information and work organization* which also mediated the indirect connections between other work community factors and *Occupational well‐being*. The clear relationship between *Information and work organization* and *Occupational well‐being* is also found in previous studies (Laine, Saaranen, et al., 2018; Van Poel et al., 2020).

The results indicated that none of the work community factors directly affect *Work ability*, which may be because occupational well‐being and work ability are close concepts containing the same elements (Cotton & Hart, 2003; Ilmarinen et al., 2008; Tengland, 2010, Laine, Saaranen, et al., 2018; Laine, Tossavainen, et al., 2018). Therefore, the strong positive direct effect of *Occupational well‐being* on *Work ability* cannot be considered a particularly surprising finding. The squared multiple correlations of *Work ability* revealed in this study indicate that the model explains *Work ability* quite well.

Unlike in the results of several previous studies (Bartels et al., 2019; Galatsch et al., 2013; Van der Heijden et al., 2017; Hirschl & Gondim, 2020; Leijon et al., 2017), the factors of *Social support* or *Influence on work shifts* did not have a significant direct relationship to *Occupational well‐being* or *Work ability* in our study. However, this result is partly supported by Ruotsalainen and others (2020) who examined various factors, such as work stressors and job control, including social support, relations to job satisfaction, stress and psychological distress in the context of Finnish home care. According to their findings, social support was not a significant predictor in any of the multivariate models. (Ruotsalainen et al., 2020.) The nature of work in home care may be relevant to these results. As occupational well‐being can be considered very context‐sensitive, the same predictors may not be universally valid in all contexts (Bartels et al., 2019). Home care can be considered fairly independent work, which reduces the significance of support and assistance from colleagues and work atmosphere. However, the standardized indirect (mediated) and standardized total (direct and indirect) effect of *Social support* on all latent variables in the model was significant, indicating that *Social support* affects *Occupational well‐being* and *Work ability* through employees' opportunities for affecting shift arrangements and *Information and work organization*. This result suggests that social support or influence on work shifts cannot alone predict occupational well‐being or work ability in the context of home care; instead, information and work organization reinforce these connections. Nevertheless, in this study, the mean value of the mean variable *Information and work organization* was the lowest. Challenges in work organization, time management and information provision in home care have also been found in earlier studies (Otto et al., 2019; Ruotsalainen et al., 2020). Van Poel and other (2020) suggest that the psychosocial work environment is essential for the work ability of nurses, highlighting mental job demands, such as time management, and participatory decision‐making to promote a beneficial working environment. According to Ruotsalainen et al. (2020), job satisfaction of home care workers could be increased by providing employees with opportunities to influence their job and manage their workdays.

The model created in this study helps target occupational well‐being and work ability development activities in home care. The importance of information provision and work organization should be emphasized, enabling home care workers to use their time optimally. This requires social support, a well‐functioning work atmosphere, and employees' opportunities to participate in and influence decision‐making in the work community (Saaranen et al., 2015). A well‐functioning community enables, for instance, changing work schedules if desired, which contributes to occupational well‐being and work ability. In addition, it is important to take into account the wishes of staff in making shift work arrangements by following equal and fair principles. (Galatsch et al., 2013).

### 5.1 Strengths and limitations

This study has several strengths. First, the hypothetical model was based on previous literature and research, which is the fundamental principle of SEM (Blunch, 2013). The two‐step approach including a validity test of the measurement model confirmed the factor structure, and the SEM model fit statistics also indicated a good model fit (Blunch, 2013; Byrne, 2010; Schreiber et al., 2006; Walker & Smith, 2017). Second, the reliability of this study is associated with material, formulation and the visual presentation of the model (see Kline, 1998). The visual presentation of the model is simple, aiming to clarify the results for the reader; non‐significant effects were deleted, resulting in providing visiblity to significant effects and squared multiple correlations (Blunch, 2013; Byrne, 2010). Finally, the questionnaire was piloted and pre‐tested for this study.

Some limitations are also noteworthy. These limitations are the response rate, relatively small sample size, cross‐sectional study design and concerns with multinormality. Although the response rate in this survey was 45%, it can be considered generally moderate (Grove et al., 2013). The study participants were given an opportunity to respond to the survey during working hours, indicating adequate representative of the target population. This makes it unlikely that only those who perceived their work ability as high responded to the survey. Moreover, this study fulfilled the widely used criterion for adequate sample sizes in EFA by Nunnally (1978), namely at least ten cases per every variable (Osborne & Costello, 2004). The model was overidentified (DF >1), which means estimated parameters exceeded the number of data points (variances and covariances) indicating an adequate sample size for SEM (Byrne, 2010). In addition, the questionnaire used in the study was fairly long, which may have influenced responding. The indirect (mediated) effects and the conclusions drawn from them must also be treated with caution because of the cross‐sectional study design. Confirmation of these results would require a longitudinal study design. As this was a study conducted in a particular municipal home care context, the results are not directly transferable to another context.

## 6 CONCLUSION

The only significant work community factor directly affecting *Occupational well‐being* was *Information and work organization*, which also mediated the indirect connections between other work community factors and *Occupational well‐being*. While *Occupational well‐being* was the only factor directly affecting *Work ability*, all work community factors indirectly affected *Work ability*. These results suggest that home care should emphasize information and work organization with optimal time use, which requires social support, a well‐functioning work atmosphere and worker's opportunities to influence and participate. Future studies should explore occupational well‐being and work ability in the context of home care.

## CONFLICT OF INTEREST

The authors declared no potential conflicts of interest with respect to the research, authorship and/or publication of this article.

## AUTHOR CONTRIBUTIONS

Vauhkonen A, Saaranen T, Honkalampi K, Järvelin‐Pasanen S, Kupari S, Tarvainen M, Perkiö‐Mäkelä M, Räsänen K and Oksanen T: Study design and/or data acquisition. Vauhkonen A, Saaranen T, Oksanen T and Perkiö‐Mäkelä M: Data analysis and interpretation. All authors have drafted the article, read the final version and approve of its publication.

## Data Availability

Research data are not shared.
